# Striving for Unity in a Culturally Fragmented World: Nested Multiple Cultural Identifications Associated With Well‐Being Through Self‐Concept Clarity

**DOI:** 10.1002/ijop.70004

**Published:** 2025-01-15

**Authors:** Simon Ozer, Seth J. Schwartz

**Affiliations:** ^1^ Department of Psychology and Behavioural Sciences Aarhus University Aarhus Denmark; ^2^ Department of Psychology University of Texas at Austin Austin Texas USA

**Keywords:** cultural identity, globalisation, multiple cultural configurations, self‐concept clarity, self‐esteem, well‐being

## Abstract

In contemporary globalised societies, global awareness and identification, as well as local and regional identifications (other than national identity), may all become increasingly important for guiding people's sense of belonging and purpose and in turn their self‐concept. As the world has become increasingly interconnected, people increasingly identify with various cultures and worldviews within both local and global contexts. Attempts to reconcile these multiple cultural identities can lead to a sense of cultural dissonance as people struggle to integrate these identities into a coherent sense of self. Accordingly, various levels/types of cultural identifications must be integrated to establish an adaptable and coherent sense of self. In two studies among participants from the United States (*N* = 754), we investigate how nested cultural identification at the state, national and global levels are associated with well‐being indirectly through self‐concept clarity and multiple cultural configurations. Results indicate that national identification is positively, and compartmentalisation negatively, associated with self‐concept clarity and in turn with well‐being. State and global cultural identifications were linked to multicultural identity integration and, indirectly, to components of well‐being. Results are discussed regarding the globalised proliferation of cultural identifications and the associated challenge of maintaining a stable and coherent sense of self.

Cultural identification represents a fundamental element of one's self‐concept and plays a vital role vis‐à‐vis individuals' psychosocial functioning. Cultural identification encompasses a sense of belonging and attachment to a particular culture or cultural group, which in turn influences various aspects of an individual's life (Côté and Levine 2002; Hermans and Dimaggio 2007). That is, cultural affiliations are proposed as providing normative templates through a clear prototype, to diminish personal uncertainty, to offer a sense of continuity and, overall, to impart insights into how to navigate one's existence in the world (Usborne and de la Sablonnière 2014). Cultural globalisation has transformed individuals' sense of cultural belonging, often resulting in multiple cultural identifications among the general population (Ferguson, Causadias, and Simenec 2023). Globalisation and cultural fragmentation have impacted national identities in many contexts, including in the United States. A number of nested cultural identities (e.g., local, state/provincial and global identifications) have become prominent (Ozer, Gamsakhurdia, and Schwartz 2024; Savage, Bagnall, and Longhurst 2005), and individuals currently navigate multiple identifications developed at the intersections among diverse cultural influences. These expansive changes in the identifications that people often must navigate reflect the evolving nature of identity in our interconnected world (Côté & Levine 2002).

The sociocultural embeddedness of psychological development suggests that self‐conception and identity are not formed in a vacuum but rather are shaped by the social and cultural contexts in which individuals live. Within this framework, the significance of sameness and coherence in identity formation across time and settings has long been acknowledged (Erikson 1968). That is, the presence of a coherent and consistent sense of identity provides a strong foundation through which individuals can develop a clear and well‐defined self‐concept (Usborne and Taylor 2010). Given the tendency for researchers to attribute international migrants' and ethnic minority individuals' behavioural and developmental outcomes to cultural influences (Causadias, Vitriol, and Atkin 2018), most research on cultural identification and well‐being has been conducted among individuals from migrant and minority backgrounds. As a result, there appears to be an important gap in understanding how majority‐group members perceive and manage their diverse cultural backgrounds within the context of exposure to globalised cultural influences (Kunst et al. 2021).

Research has explored the link between cultural identity and well‐being among immigrants and, to a lesser extent, non‐immigrants (Ferguson, Causadias, and Simenec 2023; Ward and Szabó 2023). However, little attention has been directed toward how local (state/provincial), national and global cultural identifications are linked with the experience of sameness and coherence in forming a clear and consistent self‐concept. As a result, the aim of the present studies was to examine how different levels of nested cultural identification (i.e., state, national and global) predict well‐being indirectly through various cultural identity configurations (i.e., categorisation, compartmentalisation and integration) and self‐concept clarity within the US context.

## 1 Cultural Identity and Well‐Being

Cultural identity can be understood as the process of identifying with, and incorporating into one's sense of self, the values and traditions that are characteristic of the cultural communities to which one belongs. In this regard, cultural identity not only encompasses the internalisation of these values and traditions but also includes worldview beliefs and behavioural practices (Jensen, Arnett, and McKenzie 2011). Accordingly, cultural affiliations represent an integral part of one's sense of self (Schwartz et al. 2011). This inclusion of cultural identity within one's sense of self has been supported by cross‐cultural and longitudinal research, and, in turn, research has found that national identity is positively associated with well‐being (e.g., satisfaction with life and low levels of perceived stress) (Ward and Szabó 2023).

### 1.1 Globalisation, Cultural Identifications and Self‐Conception

With globalisation promoting various levels of nested sociocultural embeddedness via interactions with different cultural and social groups (e.g., local community participation and global collaboration; Bauman 1998), cultural identification may adopt a number of forms, including but not limited to the nation in which one resides. Cultural identity has often been conflated with national cultural identification, as nations have served as the most prominent markers of group identification. However, globalisation has challenged the importance ascribed to national affiliation, as national boundaries may be blurred by global economies and interdependence among nations (Hall 1997; Rosa 2013). Further, although traditional media were often limited to and tailored for people residing in specific nations or states/provinces (e.g., broadcasting in a given language and to individuals within a nation or region), social media has permitted and encouraged connections among people from different countries. National boundaries may therefore be less important than they once were. However, research examining the impact of globalisation on national identification has yielded inconsistent findings. Studies suggest that globalisation holds the potential to diminish national identification as well as to evoke perceived cultural threats, leading to defensive national identification and blind patriotism. This inconsistency likely stems from variations in which specific measures are employed and in how different segments of society (e.g., socioeconomic status, geographic location or cultural background) are affected (Ariely 2021).

It has been argued that one potential counterreaction to globalisation involves an orientation towards local and state/provincial identities, rendering the community or region where one resides as a significant marker of cultural configuration (Hall 1997; Savage, Bagnall, and Longhurst 2005). Furthermore, Arnett (2002) asserts that individuals often establish both local/national and global identities in various settings, fostering a sense of belonging to both their nation and community as well as to the global society at large. Accordingly, in times of globalisation, people may draw from values and practices at different levels of cultural identity, reflected in their self‐conception through societal involvement ranging from the local to global levels (Ozer, Gamsakhurdia, and Schwartz 2024). Cultural identities can be adaptive, helping individuals navigate various cultural settings (e.g., local, national and global). Accordingly, these identities can provide stability in one's self‐perception, thereby enhancing self‐concept clarity (Baumeister and Muraven 1996). Additionally, orientation toward multiple cultural layers involves merging affiliations from multiple levels of cultural context to generate a cohesive and unified sense of self, whereas multiple cultural identities have been linked to well‐being (Ward and Szabó 2023), and their impact may be detrimental if they are characterised by divergence and conflict (Yampolsky et al. 2016). That is, if these nested identities embody dissimilar and irreconcilable values, they may manifest as compartmentalised, thereby impairing the internal consistency of one's overall self‐concept. On the other hand, integration fosters a unified sense of identity, providing greater clarity in one's self‐concept. Such an integration within one's cultural identity configuration has been identified as an especially cognitively complex endeavour, requiring that one's cultural identities be connected and reconciled into a coherent overall sense of self (Usborne and de la Sablonnière 2014; Ward and Szabó 2023).

Self‐concept clarity has been defined as ‘the extent to which the contents of an individual's self‐concept (e.g., perceived personal attributes) are clearly and confidently defined, internally consistent, and temporally stable’ (Campbell et al. 1996, p. 141). That is, self‐concept has been understood as a multifaceted and dynamic knowledge structure consisting of traits, values and episodic and semantic memories about the self (Campbell et al. 1996). The clarity and stability of one's sense of self have been associated with well‐being (Lodi‐Smith and Crocetti 2018; Schwartz et al. 2011). Furthermore, although globalisation has encouraged pluralism that can manifest as cultural identities within the self, Campbell, Assanand, and Di Paula (2003) found self‐concept unity, rather than plurality, to be associated with well‐being. The degree of internalisation and clarity within one's cultural identifications is therefore suggested as offering a positive sense of continuity by providing clear normative templates and a point of reference, ultimately reducing personal uncertainty and enhancing well‐being (Usborne and de la Sablonnière 2014).

Accordingly, research has demonstrated that cultural identities are linked with one's self‐construal—that is, cultural identities provide norms and values against which individuals define themselves through a clear and stable self‐conception (Ozer, Lado Gamsakhurdia, and Schwartz 2024). Although cultural norms and traditions rooted in state, national and global cultural contexts provide guiding principles in life and reduces uncertainty, the degree that these identities are perceived as integrated may be particularly important vis‐à‐vis one's experience of a consistent and stable self. As a case in point, Usborne and Taylor (2010) examined various cultural groups and found that the association between (a) the extent to which one's cultural group is perceived as clearly defined and (b) one's personal well‐being (i.e., satisfaction with life, self‐esteem and low levels of negative affect) is mediated by self‐concept clarity. Additionally, Usborne and Taylor (2012) experimentally manipulated cultural identity clarity and found that, among people who strongly identify with their cultural group, cultural identity clarity significantly enhanced well‐being through decreased personal uncertainty. These effects were not present among respondents reporting low levels of identification with their cultural group.

Globalisation, along with multiple layers of sociocultural embeddedness, has led to an increase in the number of cultural identities that individuals draw from for guidance, values and a framework for understanding themselves in contemporary society (Jensen, Arnett, and McKenzie 2011). Whereas such cultural affiliations can enhance psychological well‐being, this effect may be most likely to occur in scenarios where one's cultural affiliations work in harmony rather than conflicting with one another (Ward and Szabó 2023). However, achieving such harmony can be increasingly challenging in contemporary globalised societies (Usborne and de la Sablonnière 2014). Yampolsky and colleagues (2016) describe three ways in which multiple cultural identities are configured within one's self‐concept. *Categorisation* refers to the cognitive strategy of identifying with one cultural group over others, perhaps providing a simpler way to define oneself. *Compartmentalisation* refers to cultural identities that are kept in isolation from one another within the self, resulting in a dynamic cultural identification that manifests differently depending on the specific cultural context in which one is presently embedded. Finally, *integration* refers to the process of cohesively connecting and reconciling one's cultural identities within the self, thereby providing the possibility of simultaneously identifying with different cultural streams. Whereas compartmentalisation has been negatively associated with cultural identity clarity and well‐being, integration has been positively linked with well‐being (Yampolsky et al., 2016). Research has identified different ways of internalising cultural configurations as pathways through which cultural and ethnic identities relate to well‐being. Specifically, if one perceives one's various identities as distinct from one another, the experience of the relationships between and among these identities becomes pivotal for flourishing. In this context, multiple cultural identities serve as ingredients within identity configurations and, in turn, influence self‐concept clarity. The intervening role of how cultural identities are configured is supported by a longitudinal study (Ferrari et al. 2015), examining the role of bicultural identity integration in mediating the association of ethnic and national identities with well‐being among Italian transracial adoptees.

### 1.2 Cultural Identification in the United States

As global interconnectivity continues to increase, a significant concept gaining influence in research is the emergence of a broader sense of ‘human’ identification and the exchange of global cultural elements (e.g., fashion and popular entertainment). Such global identification has been associated with individual differences (e.g., empathy, openness and authoritarianism), socialisation, education and perceived positive evaluation of global identity by significant others (McFarland et al. 2019). Within the specific context of the United States, a strong sense of nationality and national culture has been acknowledged as potentially unifying people from different ethnic and religious backgrounds (Schildkraut 2014). Nevertheless, within such a large, diverse and highly populated country, regional differences are likely to emerge—Woodard (2012) has argued that 11 sub‐national cultural regions exist within the US. For example, the Deep South is more collectivistic, the Mountain West is grounded in rugged individualism and the Northeast is highly cosmopolitan and dominated by large cities such as New York, Boston and Washington DC. As a result, the United States is simultaneously (a) a single nation based on ideals of individual freedom and responsibility and (b) a somewhat self‐contradictory collection of states and regions whose core value systems differ sharply from one another (e.g., with regard to reproductive rights and gun control). A sense of belonging to one's state or region, as well as to the country as a whole, might be integrated as an important component of the self. Texans, New Yorkers and Californians, for example, may derive a great deal of pride from living in these states *as well as* from being American. Somewhat similar findings have emerged in the UK—local or regional identities have emerged as particularly important because they often include kinship ties and emotional connections with specific locations (Savage, Bagnall, and Longhurst 2005).

Although globalisation has, to some extent, challenged the concept of strong nation states and associated national identities (Bauman 1998; Hall 1997), regional or state affiliation may emerge as a central cultural affiliation for some and, likewise, global identification may emerge as an increasingly salient and relevant cultural identification. As noted above, acculturation research has extended the conception of cultural identity to include global identifications rooted in globalisation (Ward and Szabó 2023). Although state/provincial, national and global cultural identifications represent different levels of cultural context, they do not necessarily emerge in a conflictual and compartmentalised forms within the self but may, in some cases, be nested and integrated so as to contribute to a clearer sense of who one is and, in turn to promote well‐being.

### 1.3 The Current Studies

Drawing on the work of Usborne and Taylor (2010) and Usborne and de la Sablonnière (2014) linking cultural identity clarity with well‐being through self‐concept clarity, the aim of the present studies was to examine how specific levels of cultural identification (i.e., state, national and global), along with the configuration (i.e., categorisation, compartmentalisation and integration) of such identifications within the US context, relate to well‐being (as indexed by self‐esteem, perceived stress and flourishing) both directly and indirectly through a clear, confident and coherent understanding of oneself (i.e., self‐concept clarity). That is, whereas cultural identity clarity refers to the extent to which individuals understand, and are confident in, their beliefs about their cultural affiliations, here we examine how specific *nested* cultural identities, and their configurations, are associated with a clearer self‐concept (Ozer, Gamsakhurdia, and Schwartz 2024). Accordingly, our current study advances the literature by focusing specifically on cultural identifications that are nested within another—such as Miami resident, Floridian and American. Especially given the federalism within the USA. Constitution and the fact that states often diverge from one another politically, socially and culturally, state‐level identification may emerge as an important predictor.

Based on a pilot project indicating that the three levels of cultural identity were directly associated with well‐being and indirectly linked with well‐being through self‐concept clarity (see the Supporting Information), we tested the following three hypotheses across two studies: (H1) state, national and global cultural identifications would be positively and directly associated with well‐being^1^; (H2) state, national and global cultural identifications would be positively and indirectly associated with well‐being through self‐concept clarity; and we expected that the three cultural identities would be associated with well‐being through the ways these cultural identities are internalised. Specifically, we expected (H3) that multicultural identity integration would be positively associated with well‐being through self‐concept clarity, whereas compartmentalisation and categorisation would be negatively associated with well‐being through self‐concept clarity. In Study 1, we tested H1 and H2. As an exploratory aim, we complemented our variable‐centred approach with an exploratory post hoc person‐centred approach to examine how dissimilar cultural identity patterns relate to self‐concept clarity and well‐being. In Study 2, we extended the results of Study 1 by including a measure of multicultural identity configuration (categorisation, compartmentalisation and integration) to test H3.

## 2 Study 1

In the first study (preregistration: https://aspredicted.org/JH3_PLL ^2^), we addressed the limitations of the pilot study through replication and by including a broader measure of cultural identification, the complete Self‐Concept Clarity Scale, two measures of well‐being (flourishing and self‐esteem) and a measure of perceived stress.

### 2.1 Procedure

Data were collected as part of larger studies^3^ through an online questionnaire. Participants were recruited from Prolific, and the questionnaire took the participants an average of 9 min and 12 s to complete. Participants from the pilot study were excluded from participating in Study 1. As an inclusion criterion, participants had to be US citizens.

### 2.2 Participants

We aimed for a sample size above 300 based on recommendations regarding adequate statistical power for SEM analysis (Kline 2015). Participants were 323 US citizens (eight cases were deleted because they completed less than half of the study survey). The participants' mean age was 42.87 (*SD* = 14.03; all above 18 years old), and 50.2% were male, 48.9% were female and 0.9% indicated other. With regard to ethnicity, 70.9% identified as White, 11.5% as Black, 8.0% as Hispanic, 5.9% as Asian and 3.7% as other. Participants resided in various states, with most living in Florida (12.3%), Texas (10.2%), Pennsylvania (6.2%), Ohio (5.0%) and Virginia (4.3%).

### 2.3 Measurement

Demographic information concerning age, gender, ethnicity and state of residence was collected first. Three‐item measures of cultural identification were used for each relevant cultural stream referring to the context of the United States (i.e., state, *α* = 0.92; nation *α* = 0.92; global, *α* = 0.94). The items were developed from the Multigroup Ethnic Identity Measure (Phinney 1992), reflecting identification, cultural attachment and valence toward the cultural group in question (state, nation or global community). Sample items read: ‘I identify with the local culture of the state that I live in’, ‘I feel a strong attachment toward the American culture’ and ‘I feel good about the global culture’. These items were answered using a 7‐point Likert scale ranging from 1 (*Strongly disagree*) to 7 (*Strongly agree*).

Self‐concept clarity was assessed using the Self‐Concept Clarity Scale (Campbell et al. 1996; *α* = 0.93) consisting of 12 items. A sample item reads: ‘I seldom experience conflict between the different aspects of my personality’. These items were answered using a 5‐point Likert scale ranging from 1 (*Strongly disagree*) to 5 (*Strongly agree*).

Self‐esteem was measured through the Rosenberg Self‐Esteem Scale (Rosenberg 1965; *α* = 0.93) and measures both positive and negative feelings about the self through 10 items about the participant's current feelings about himself or herself. A sample item reads, ‘I feel I do not have much to be proud of’ (reverse scored). Responses were provided on a 7‐point Likert scale ranging from 1 (*Strongly agree*) to 7 (*Strongly disagree*).

The Perceived Stress Scale (Cohen, Kamarck, and Mermelstein 1983; *α* = 0.90) was used to measure participants' stress levels by tapping into experiences of unpredictability, uncontrollability and overload in one's life during the month prior to assessment. The scale consists of 10 items, including: ‘In the last month, how often have you found that you could not cope with all the things that you had to do?’ Responses were provided on a 5‐point Likert scale ranging from 1 (*Never*) to 5 (*Very often*).

Flourishing was assessed using the Flourishing Scale (Diener et al. 2010; *α* = 0.92), which consists of eight items tapping into self‐perceived success in important areas such as relationships, self‐esteem, purpose and optimism. Sample items include ‘I lead a purposeful and meaningful life’. Responses were provided on a 7‐point Likert scale ranging from 1 (*Strongly disagree*) to 7 (*Strongly agree*).

### 2.4 Analytic Plan

All statistical analyses were conducted in SPSS v.29 and Mplus 8.8 (Muthén and Muthén 1998–2011). The hypothesised indirect effects model was examined using structural equation modelling (SEM) employing maximum likelihood estimation with robust standard errors (MLR). Kline's (2015) criteria for evaluating model fit were used, suggesting that the following fit index cut‐offs should be satisfied: comparative fit index (CFI) ≥ 0.90, standardised root‐mean‐square residual (SRMR) ≤ 0.08 and root‐mean‐square error of approximation (RMSEA) ≤ 0.08.

### 2.5 Results

The correlation matrix (Table 1) yields some results that diverge from those found in the pilot study. In terms of identification, only national cultural identity was significantly linked with self‐concept clarity, and global identity was not significantly associated with either self‐esteem or perceived stress.

**TABLE 1:** Correlation matrix and means for Study 1.

	2.	3.	4.	5.	6.	7.	*M* (*SD*)
1. State identification	0.74**	0.18**	0.11	0.28**	−0.29**	0.42**	4.24 (1.59)
2. National identification		0.17**	0.18**	0.32**	−0.33**	0.41**	4.42 (1.61)
3. Global identification			−0.04	0.09	−0.09	0.21**	4.16 (1.43)
4. Self‐concept clarity				0.64**	−0.57**	0.40**	3.54 (0.96)
5. Self‐esteem					−0.69**	0.77**	4.97 (1.34)
6. Perceived stress						−0.61**	2.72 (0.78)
7. Flourishing							5.10 (1.23)

*Note*: *p < 0.05; **p < 0.01.

**FIGURE 1:**
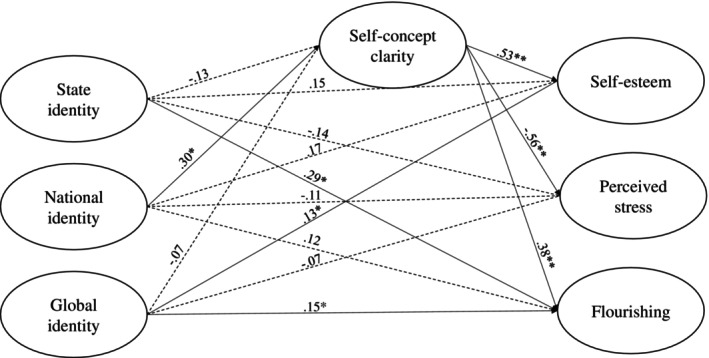
The results of the indirect effects model from Study 1. *marks *p* < 0.01 and **marks *p* < 0.001.

To test H1, which states that national and global cultural identifications would be positively and directly associated with well‐being, we estimated direct effects within our model. To test H2, which proposed that cultural identities would be positively associated with well‐being through self‐concept clarity in our model, we estimated indirect effects within our indirect effects model. This hypothesised indirect effects model was estimated using SEM, employing latent variables (state, national and global identifications) or utilising parcelling (self‐concept clarity, self‐esteem, perceived stress and flourishing) to model single variables as latent constructs. The model yielded acceptable fit to the data, *χ* ^2^(208) = 588.11, *p* < 0.001; CFI = 0.936; SRMR = 0.057; RMSEA = 0.075, 90% CI = [0.068; 0.082]. Results (Figure 1) partly supported H1, as state cultural identification was only linked directly with one aspect of well‐being (flourishing), and global cultural identification was directly associated with both self‐esteem and flourishing.

Specifically, results indicated a positive association between national cultural identification and self‐concept clarity (*β* = 0.30, *p* = 0.008, 95% CI = [0.08; 0.53]) but no associations of state (*β* = −0.13, *p* = 0.274, 95% CI = [−0.35; 0.10]) or global (*β* = −0.07, *p* = 0.263, 95% CI = [−0.19; 0.05]) identification with self‐concept clarity. Global cultural identification (*β* = 0.13, *p* = 0.005, 95% CI = [0.04; 0.23]) and self‐concept clarity (*β* = 0.53, *p* < 0.001, 95% CI = [0.41; 0.65]), but not state (*β* = 0.15, *p* = 0.098, 95% CI = [−0.03; 0.33]) or national (*β* = 0.17, *p* = 0.077, 95% CI = [−0.02; 0.35]) cultural identifications, were significantly and positively linked with self‐esteem. Additionally, self‐concept clarity (*β* = −0.53, *p* < 0.001, 95% CI = [−0.66; −0.46]), but neither state (*β* = −0.14, *p* = 0.139, 95% CI = [−0.33; 0.05]), national (*β* = −0.11, *p* = 0.247, 95% CI = [−0.29; 0.07]), nor global (*β* = −0.07, *p* = 0.173, 95%CI = [−0.16; 0.03]) cultural identification, was significantly and negatively linked with perceived stress. Moreover, state (*β* = 0.29, *p* = 0.002, 95% CI = [0.11; 0.47]) and global (*β* = 0.15, *p* = 0.002, 95% CI = [0.06; 0.25]) cultural identifications, as well as self‐concept clarity (*β* = 0.38, *p* < 0.001, 95% CI = [0.27; 0.48]), but not national cultural identification (*β* = 0.12, *p* = 0.197, 95% CI = [−0.06; 0.31]), were significantly and positively associated with flourishing.

Partly supporting H2, we found three significant indirect paths relating national cultural identification positively with self‐esteem (*β* = 0.16, *p* = 0.014, 95% CI = [0.05; 0.29]) and flourishing (*β* = 0.12, *p* = 0.015, 95% CI = [0.02; 0.21]) and negatively with perceived stress (*β* = −0.17, *p* = 0.010, 95% CI = [−0.28; −0.04]) through self‐concept clarity. However, state and global cultural identities were not significantly associated with the three aspects of well‐being through self‐concept clarity.

We then sought to complement our variable‐centred approach with an exploratory post hoc person‐centred approach. We conducted a two‐step cluster analysis in SPSS. Employing Schwarz's Bayesian Information Criterion for determining the number of clusters, we found two clusters based on the nine cultural identity items (i.e., three items apiece for state, national and global identities). These two clusters reflected strong (62.8%) and weak (37.2%) cultural identifications. Results indicated that national and state cultural identities were associated with high predictor importance (i.e., significance of specific variables) (≥ 0.73), whereas global identification had low predictor importance (≤ 0.03). Mean comparisons (see Table 2 for means and statistical tests) across the two clusters indicated that participants in the strong cultural identity configuration cluster scored significantly higher on state (*p* < 0.001), national (*p* < 0.001) and global (*p* = 0.021) cultural identities, as well as self‐concept clarity (*p* = 0.003), self‐esteem (*p* < 0.001) and flourishing (*p* < 0.001), compared to the weak cultural identity configuration cluster. Moreover, the weak cultural identity configuration cluster scored significantly higher on perceived stress compared to the strong cultural identity cluster (*p* < 0.001).

**TABLE 2:** Means and mean comparisons across the two identity configuration clusters.

	Strong identity configuration M (*SD*)	Weak identity configuration M (*SD*)	Statistical comparison
State identification	5.14 (1.08)	2.72 (1.09)	*t* (321) = 19.39, *p* < 0.001, *d* = 2.232
National identification	5.37 (1.01)	2.80 (1.05)	*t* (321) = 21.74, *p* < 0.001, *d* = 2.504
Global identification	4.30 (1.43)	3.92 (1.40)	*t* (321) = 2.32, *p* = 0.021, *d* = 0.267
Self‐concept clarity	3.66 (0.93)	3.34 (0.98)	*t* (321) = 2.96, *p* = 0.003, *d* = 0.341
Self‐esteem	5.23 (1.23)	4.51 (1.41)	*t* (321) = 4.80, *p* < 0.001, *d* = 0.552
Perceived stress	2.56 (0.72)	3.01 (0.80)	*t* (321) = 5.20, *p* < 0.001, *d* = 0.599
Flourishing	5.41 (1.07)	4.57 (1.32)	*t* (321) = 6.21, *p* < 0.001, *d* = 0.715

### 2.6 Preliminary Discussion

Study 1 only partly replicated the findings of the pilot study, as only national cultural identification was indirectly linked with well‐being through self‐concept clarity (H2); state cultural identification was only linked directly with one aspect of well‐being (flourishing); and global cultural identification was directly associated with both self‐esteem and flourishing (H1). Accordingly, national cultural identity appears to be influential regarding self‐conception and, in turn, well‐being. Furthermore, the three cultural identities appear to be reconcilable, as suggested by the strong cultural identity configuration cluster being positively related to well‐being and self‐concept clarity. These findings indicate that cultural identity represents a psychologically promotive factor.

Our findings suggest that a strong endorsement of state, national and global cultural identities represent the most adaptive identity profile. However, clustering people with strong state, national and global cultural identities does not indicate the degree to which those identities are integrated. Accordingly, research is needed to examine how and how well these cultural identifications can be simultaneously internalised and integrated to provide unified cultural identities (Gardner and Garr‐Schultz 2017). For example, the low predictor importance of global identification vis‐à‐vis the cultural identity clusters might reflect the ambiguity of the concept of ‘global culture’. Accordingly, additional research is needed to examine the effects of different ways of internalising nested cultural configurations.

## 3 Study 2

In the second study (preregistration: https://aspredicted.org/BRR_5RJ), we addressed the limitations of the previous study by replicating and extending Study 1. This extension involved assessing how the three nested cultural identities might be internalised—specifically, through categorisation, compartmentalisation or integration. Accordingly, we explored the significance of one's cultural configuration in relation to self‐concept clarity and well‐being.

### 3.1 Procedure

New data were collected through an online questionnaire, and participants were recruited from Prolific. The questionnaire took the participants an average of 13 min and 26 s to complete. Participants from the pilot study and from Study 1 were excluded from participating in Study 2. As in the previous studies, an inclusion criterion was that participants had to be US citizens.

### 3.2 Participants

We aimed for a sample size well beyond 300, aligning with the guidance for robust statistical power for SEM (Kline, 2015), especially given the increased complexity of our indirect effects model compared over the model estimated in Study 1. Participants were 431 US citizens (after deleting 20 responses that were less than 50% complete; no participants failed both attention checks). The participants' mean age was 42.93 (*SD* = 13.80), and 49.0% were male, 49.0% were female, 1.9% indicated other and 0.2% did not answer that question. With regard to ethnicity, 75.6% were identified as White, 12.8% as Black, 6.0% as Asian, 3.7% as Hispanic and 1.9% as other. Participants resided in various states, with most living in Florida (7.4%), Texas (7.2%), Pennsylvania (6.7%), California (5.8%) and Michigan (5.6%).

### 3.3 Measurement

Demographic information concerning age, gender, ethnicity and state of residence was collected first. The same measures as employed in Study 1 were included: cultural identification (i.e., state, *α* = 0.92; nation *α* = 0.92; global, *α* = 0.91), Self‐Concept Clarity Scale (Campbell et al. 1996; *α* = 0.93), Rosenberg Self‐Esteem Scale (Rosenberg 1965; *α* = 0.94), Perceived Stress Scale (Cohen, Kamarck, and Mermelstein 1983; *α* = 0.90) and Flourishing Scale (Diener et al. 2010; *α* = 0.93). Additionally, multicultural identity configuration was assessed by the Multicultural Identity Integration Scale (Yampolsky et al. 2016). The scale assesses three types of multiple cultural configurations: categorisation (e.g., ‘I identify exclusively with one culture’; *α* = 0.81), compartmentalisation (e.g., ‘Each of my cultural identities is a separate part of who I am’; *α* = 0.91) and integration (e.g., ‘The differences between my cultural identities complete each other’; *α* = 0.91). Participants were asked to answer with regard to state, national and global cultural identities, and responses were recorded on a 7‐point Likert scale ranging from 1 (*Not at all*) to 7 (*Exactly*).

### 3.4 Results

The correlation matrix (Table 3) yields some results that diverge from those found in Study 1. In terms of identifications, both state and national cultural identities were significantly linked with self‐concept clarity, but global identity was not significantly associated with categorisation or with self‐concept clarity. Aside from this one nonsignificant association, cultural identities were positively associated with the three aspects of multicultural identity, whereas compartmentalisation was negatively, and integration positively, associated with self‐concept clarity.

**TABLE 3:** Correlation matrix and means for Study 2.

	2.	3.	4.	5.	6.	7.	8.	9.	10.	M (*SD*)
1. State identification	0.66**	0.37**	0.23**	0.18**	0.33**	0.18**	0.34**	−0.32**	0.46**	4.27 (1.56)
2. National identification		0.28**	0.20**	0.20**	0.27**	0.24**	0.32**	−0.36**	0.39**	4.37 (1.60)
3. Global identification			0.07	0.17**	0.32**	0.03	0.17**	−0.13**	0.31**	4.21 (1.41)
4. Categorisation				0.61**	0.17**	−0.02	0.02	−0.04	0.09	3.13 (1.38)
5. Compartmentalisation					0.19**	−0.17**	−0.05	0.07	0.05	2.74 (1.26)
6. Integration						0.10*	0.27**	−0.17**	0.33**	4.24 (1.31)
7. Self‐concept clarity							0.58**	−0.58**	0.37**	3.55 (0.95)
8. Self‐esteem								−0.74**	0.78**	4.95 (1.45)
9. Perceived stress									−0.61**	2.72 (0.77)
10. Flourishing										5.16 (1.26)

*Note*: *p < 0.05; **p < 0.01.

**FIGURE 2:**
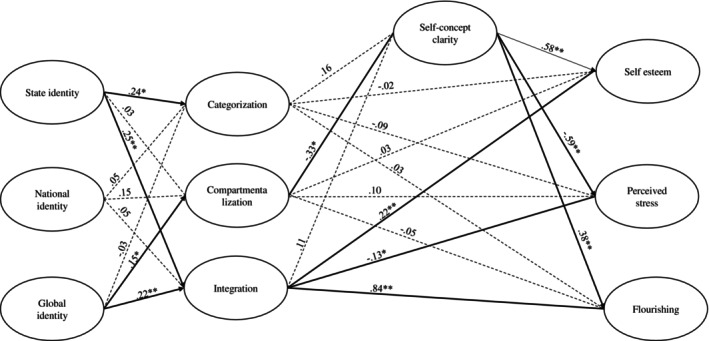
The results of the indirect effects model from Study 2. *marks *p* < 0.01 and **marks *p* < 0.001.

The hypothesised indirect effects model associating cultural identities with self‐esteem, perceived stress and flourishing through multicultural identity configurations and self‐concept clarity was estimated using SEM, utilising latent variables (for state, national and global identifications) or using parcelling (for multiple cultural configurations, self‐concept clarity, self‐esteem, perceived stress and flourishing) to model latent variables. This model yielded acceptable fit to the data, *χ* ^2^(471) = 858.67, *p* < 0.001; CFI = 0.961; SRMR = 0.075; RMSEA = 0.044, 90% CI = [0.039; 0.048]. Results (Figure 2) indicated a positive association between state cultural identification and categorisation (*β* = 0.24, *p* = 0.007, 95% CI = [0.07; 0.41]) but no associations of national (*β* = 0.05, *p* = 0.606, 95% CI = [−0.13; 0.23]) or global (*β* = −0.03, *p* = 0.655, 95% CI = [−0.16; 0.10]) identifications with categorisation. Moreover, we found a positive association between global cultural identification and compartmentalisation (*β* = 0.15, *p* = 0.007, 95% CI = [0.04; 0.25]) but no associations of state (*β* = 0.03, *p* = 0.759, 95% CI = [−0.14; 0.19]) or national (*β* = 0.15, *p* = 0.057, 95% CI = [−0.01; 0.31]) identifications with compartmentalisation. Further, state (*β* = 0.25, *p* < 0.001, 95% CI = [0.13; 0.36]) and global (*β* = 0.22, *p* < 0.001, 95% CI = [0.13; 0.31]) cultural identifications were positively linked with integration, whereas national cultural identity was not (*β* = 0.05, *p* = 0.425, 95% CI = [−0.07; 0.16]).

We found a negative association between compartmentalisation and self‐concept clarity (*β* = −0.33, *p* = 0.002, 95% CI = [−0.54; −0.12]) but no associations of categorisation (*β* = 0.16, *p* = 0.122, 95% CI = [−0.04; 0.36]) or integration (*β* = 0.11, *p* = 0.070, 95% CI = [−0.01; 0.23]) with self‐concept clarity. Moreover, self‐concept clarity (*β* = 0.58, *p* < 0.001, 95% CI = [0.47; 0.69]) and integration (*β* = 0.22, *p* < 0.001, 95% CI = [0.13; 0.30]) were positively linked with self‐esteem, whereas categorisation (*β* = −0.02, *p* = 0.841, 95% CI = [−0.16; 0.13]) and compartmentalisation (*β* = 0.03, *p* = 0.717, 95% CI = [−0.12; 0.18]) were not. Similarly, self‐concept clarity (*β* = −0.59, *p* < 0.001, 95% CI = [−0.69; −0.49]) and integration (*β* = −0.13, *p* = 0.010, 95% CI = [−0.22; −0.03]) were negatively linked with perceived stress, whereas categorisation (*β* = −0.09, *p* = 0.256, 95% CI = [−0.24; 0.06]) and compartmentalisation (*β* = 0.10, *p* = 0.237, 95% CI = [−0.06; 0.26]) were not. Finally, self‐concept clarity (*β* = 0.38, *p* < 0.001, 95% CI = [0.25; 0.51]) and integration (*β* = 0.84, *p* < 0.001, 95% CI = [0.56; 1.13]) were positively associated with flourishing, whereas categorisation (*β* = 0.03, *p* = 0.803, 95% CI = [−0.18; 0.23]) and compartmentalisation (*β* = −0.05, *p* = 0.654, 95% CI = [−0.27; 0.17]) were not.

When we tested H3, we found three significant indirect paths relating compartmentalisation negatively with self‐esteem (*β* = −0.19, *p* = 0.003, 95% CI = [−0.32; −0.07]) and flourishing (*β* = −0.13, *p* = 0.007, 95% CI = [−0.22; −0.03]) and positively with perceived stress (*β* = 0.20, *p* = 0.003, 95% CI = [0.07; 0.32]) through self‐concept clarity. Accordingly, only the effects of compartmentalisation on well‐being through self‐concept clarity supported our third hypothesis.

### 3.5 Preliminary Discussion

Based on our results for Study 2, the mean for multicultural integration (*M* = 4.24, *SD* = 1.31) was greater than the mean for compartmentalisation (*M* = 2.74, *SD* = 1.26). Based on strong correlations, it seems that local and national cultural identities might be particularly integrated. Accordingly, the three nested cultural identities appear to be more unified than separated in our sample. However, state identity was only modestly associated with categorisation and compartmentalisation, and global identity was only modestly associated with integration. These findings may reflect some degree of individual differences in how these identities are configured (e.g., some people shift between global and state cultures, whereas others are able to integrate these identities). Integration and self‐concept clarity were only weakly correlated, suggesting that other factors, such as the strength of the three identities, may be more relevant vis‐a‐vis self‐concept. Surprisingly, only compartmentalisation was significantly associated with self‐concept clarity and indirectly with well‐being, whereas a direct association emerged between integration and well‐being that did not operate indirectly through self‐concept clarity.

## 4 General Discussion

Globalisation has introduced new sets of cultural identity configurations and, in turn, has impacted people's sense of self. Additionally, many nations are divided into states or provinces that are often quite different from one another and with which individuals can also identify. This scenario therefore provides individuals with at least three levels of collective identification—state/provincial, national and global.

Our first hypothesis expecting state, national and global cultural identifications to be positively and directly associated with well‐being was only partly supported in Study 1 (state and global cultural identities). However, our second hypothesis suggesting that state, national and global cultural identifications would be positively and indirectly associated with well‐being through self‐concept clarity was supported only for national identification in Study 1. Our third hypothesis was only partly supported, as compartmentalisation was negatively and indirectly associated with well‐being through self‐concept clarity, whereas integration was directly linked with well‐being in Study 2. These results, together with our findings from Study 1 that a generally high cultural identification is linked to self‐concept clarity and well‐being, are aligned with previous results linking cultural identity clarity positively, and compartmentalisation negatively, with well‐being through self‐concept clarity (Ozer, Lado Gamsakhurdia, and Schwartz 2024; Usborne and Taylor 2010, Yampolsky et al. 2016).

### 4.1 Local Identification, Global Identification and Stable Self‐Perception

In Study 1 (as well as in the pilot study), national cultural identification emerged as an important predictor of self‐concept clarity. This pattern of findings suggests that national cultural values and behaviours play a significant role in shaping individuals' sense of self. Additionally, these results imply that individuals tend to delineate and internalise their identification with their national culture into their sense of self, whereas state and global identifications are less strongly associated with one's overall sense of self. One explanation for this pattern of results could be that a primary national cultural identity provides greater and more adaptive self‐concept unity than a pluralism of cultural identities (Campbell, Assanand, and Di Paula 2003), although this might vary regarding the homogeneity of the specific state culture (e.g., people in New York, California and Texas might hold a stronger sense of state culture compared to other contexts, such as Florida, where many people moved from somewhere else). Moreover, clear collective norms, as presented by national‐level culture, are necessary for achieving cultural identity clarity, which might not be the case for less delineated levels of cultural demarcation reflected in state and (especially) global cultural streams (Gardner and Garr‐Schultz 2017). Our results indicate that the national level of rootedness still appears to be highly relevant and influential in times of globalisation, providing a sense of self and stability that promotes well‐being. These findings comport with similar research from Denmark, where national, but not global, identification was linked with self‐concept clarity. In this context, a third cultural identification—European identity—was negatively linked with self‐concept clarity, reflecting possible ambiguity and conflict between the cultural positions within the self (Ozer, Lado Gamsakhurdia, and Schwartz 2024).

However, in Study 2, national identity did not emerge as important vis‐à‐vis multiple cultural identity configurations. Rather, state cultural identity was associated with categorisation, possibly reflecting a predominant cultural identification at the state level. Both state and global cultural identifications were related to integration, perhaps reflecting possibilities of including local and global identities in a non‐conflictual multicultural configuration. Furthermore, state and global identifications may reflect a potentially dialectical relationship between local and global levels of identity, suggesting that these levels of cultural affiliation are not detached but rather are shaped in reaction to each other (e.g., the local can emerge as a defensive response to the global or the global can be perceived as a local awareness of the world; Savage, Bagnall, and Longhurst 2005). Accordingly, global identity was linked with compartmentalisation, which was further negatively associated with self‐concept clarity. This set of findings may reflect challenges of reconciling globalised cultural identities, which may potentially result in cultural identity confusion (Arnett 2002).

Global culture has been conceptualised as comprising cultural elements originating from various sociocultural contexts (Jensen, Arnett, and McKenzie 2011). In the context of the United States, the content of all three cultural streams (state, national and global) are predominantly Western. However, the conception and content of a global cultural stream may carry different meanings in different parts of the world, potentially leading to ambiguity regarding the meaning of global identity. These nuanced cultural configurations involving state and global cultural identities highlight the importance of how cultural identity is measured (Ozer, Gamsakhurdia, and Schwartz 2024). Accordingly, our results from Study 2 indicate that global identity is positively associated with both compartmentalisation and integration. This finding suggests that global identity, being malleable, can incorporate diverse elements from local cultural aspects (e.g., state‐level reproductive rights) while also serving as a superordinate, inclusive identity that encompasses other levels of cultural identification.

### 4.2 A Stable and Coherent Sense of Self in a Changing World

In globalised and culturally heterogeneous societies, various cultural identities are often part of people's self‐concept. Although a strong cultural identity has been conceptualised as stabilising for the individual's sense of self and well‐being (Usborne and de la Sablonnière 2014), our cluster analytic results suggest that various levels of cultural identification can provide similar benefits in times of globalised sociocultural disruption (e.g., significant changes and challenges in social and cultural norms). Accordingly, the negative association between compartmentalisation and self‐concept clarity in Study 2 reflects challenges inherent in reconciling multiple cultural identities. That is, fragmentation and divergence appear to hold greater relevance for self‐concept clarity than does the integration of multiple cultural identities. Hence, a clear self‐conception can include multiple cultural identifications, even if they are not fully integrated. However, when cultural identities are compartmentalised, suggesting divergence and potential conflicts between them, such incompatibility can fragmentise one's self‐concept. This finding is aligned with previous work associating compartmentalisation with self‐concept clarity and linking integration with well‐being (Yampolsky et al. 2016).

Adopting and integrating an inclusive global cultural identity may be adaptive for navigating and internalising globalised multicultural perspectives. In a representative German sample, Kunst et al. (2023) found that global identity—but not regional and national identities—predicts a strong association between one's heritage cultural maintenance and other culture adoption, reflecting a more open‐minded integrative approach to multiculturalism. In addition, research has found that living abroad and being exposed to intercultural contact increases self‐concept clarity and, in turn, significant life decisions because it prompts self‐discerning reflections (Adam et al. 2018). Similarly, drawing on various cultural levels of cultural identification might enhance careful consideration of what these levels mean, what parts of one's self are derived from personal values and what identifications result from shared cultural beliefs. That is, whereas cultural rootedness appears to establish a sense of self and belonging in a transforming world, various cultural affiliations may help to stimulate adaptive self‐reflection. Importantly, self‐conception has been found to differ across cultural contexts (e.g., self‐conception in East Asian cultures includes more contradictions; DeMarree and Bobrowski 2018). Accordingly, caution should be included when generalising our findings to other national contexts because less self‐concept clarity may be adaptive in some Eastern globalised contexts.

## 5 Limitations

It is important to interpret the present findings in light of some limitations. Firstly, the cross‐sectional design restricts our ability to establish directional or causal relationships. Further research utilising longitudinal designs would be valuable in determining the directionality of the associations we observed, as well as in comparing different developmental sequences (e.g., Schwartz et al. 2011). Secondly, our assessment of state identity refers to the cultural context of the state in which the person currently resides and does not account for intranational migration (e.g., New Yorkers moving to Florida or Californians moving to Texas). Moreover, the concept of global culture is somewhat ambiguous, leading to potential variations in interpretation among participants. For instance, some may view global culture as encompassing elements such as Hollywood movies, mindfulness and Pokémon, whereas others may see it as a shared identification transcending national boundaries. Future research should delve into the phenomenological understandings of these diverse cultural streams. Thirdly, our assessment of cultural identification did not include additional cultural identifications (e.g., ethnic groups). Future research could investigate how majority‐group members' cultural identification may differ from those of minority cultural groups (i.e., including ethnic cultural identification such as being Hispanic or religious identification such as being Jewish). Fourthly, the salience and characteristics of state identity may differ across the US (e.g., Texas was once an independent country; please see the Supporting Information for an investigation of this).

## 6 Conclusion

The rise of globalisation, along with the proliferation of cultural affiliations, has highlighted the importance of establishing a coherent sense of self in a changing world. One's sense of self is developed within sociocultural contexts and in relation to various group memberships. In the present studies, we found that national cultural identification was associated with well‐being indirectly through self‐concept clarity. Although state and global cultural identifications were directly linked with aspects of well‐being, these cultural affiliations were not associated with establishing a clear sense of self. Nevertheless, state and global cultural identifications were significantly associated with the three multicultural identity configurations. Although globalisation has challenged the importance of national cultural identifications, our results suggest that national identity remains associated with a coherent self‐concept in today's globalised world. Nevertheless, our research points toward the adaptive complexity of contemporary globalised cultural identification, necessitating affiliation with and belonging to various cultural contexts including the local, national and global. We hope that the present study inspires further work in this direction.

## Ethics Statement

The present study adhered to the country of the author's national ethical guidelines for research as well as being in accordance with the ethical standards as set forth in the 1964 Declaration of Helsinki and its later amendments. Informed consent was obtained from all individual participants who were included in the study.

## Conflicts of Interest

The authors declare no conflicts of interest.

## Supporting information


Data S1.

## Endnotes

^1^
In addition to the pre‐registered analyses, we also examined both direct associations between cultural identity and well‐being as well as indirect associations through self‐concept clarity.

^2^
In a departure from the pre‐registered analysis, self‐esteem was incorporated as an outcome variable instead of self‐efficacy as a mediating variable in order to capture a broader spectrum of psychological well‐being, aligned with the study by Usborne and Taylor (2010).

^3^
The data sets were part of larger studies. Some measures were used in another unrelated study to address a different research question regarding the development of a social psychological measurement scale (please see the Supporting Materials).
