# Supplementary material for: Striving for Unity in a Culturally Fragmented World: Nested Multiple Cultural Identifications Associated With Well‐Being Through Self‐Concept Clarity
Source: Int J Psychol. 2025 Jan 15;60(1):e70004. doi: 10.1002/ijop.70004 (PMC11733707; doi:10.1002/ijop.70004)
Supplement: Supplementary file 1 — Data S1. [file IJOP-60-e70004-s001.docx]

Supplementary Material

**Striving for Unity in a Culturally Fragmented World: Nested Multiple Cultural Identifications Associated with Well-Being through Self-Concept Clarity**

**Content**

Items used for measuring cultural identity..….…….………………………………………………………………2

Additional Measures included in the Data Collection………………………………….…………………………..3

Pilot study…………...…………………………………….………………………………………………….….…5

Examining state identity……………………………………………………………………………………………9

References…………………………………………………………………………………………………………..10

**Items used for measuring cultural identity (Item 1 was utilized in the Pilot Study and all three items were included in Studies 1 and 2).**

State cultural identity

| 1) I identify with the local culture of the state that I live in.  2) I feel a strong attachment toward the local culture of the state that I live in.  3) I feel good about the local culture of the state that I live in. |
| --- |

National cultural identity

| 1) I identify with American culture.  2) I feel a strong attachment toward the American culture.  3) I feel good about the American culture. |
| --- |

Global cultural identity

| 1) I identify with global culture.  2) I feel a strong attachment toward the global culture.  3) I feel good about the global culture. |
| --- |

**Measures included in the Data Collection**

For the sake of transparency, we list each measure included in the data collections for both this project (black text) and another project (red text) in the order they were presented to the participants. Moreover, we indicate which measures are included in both research projects (green text).

Pilot Study:

21 items pertaining to the development of a revised measure of the theoretical concept of life attachment.

Cultural identity (local, national, and global)

One item from the Self-Concept Clarity Scale (Campbell et al., 1996)

Measures employed in both the present study and other study: Satisfaction with Life Scale (Diener et al., 1985).

Study 1:

Life Attachment Scale – Revised

Openness to the Future Scale (Botella et al., 2018)

Life Attachment Scale (Ozer & Bertelsen, 2019)

Cultural identity (local, national, and global)

Self-Concept Clarity Scale (Campbell et al., 1996)

Rosenberg Self-Esteem Scale (Rosenberg, 1965)

Measures employed in both the present study and other study: The Perceived Stress Scale (Cohen et al., 1983), Flourishing Scale (Diener et al., 2009).

Study 2:

Life Attachment Scale – Revised

Openness to the Future Scale (Botella et al., 2018)

Life Attachment Scale (Ozer & Bertelsen, 2019)

Cultural identity (local, national, and global)

Self-Concept Clarity Scale (Campbell et al., 1996)

Rosenberg Self-Esteem Scale (Rosenberg, 1965)

Measures employed in both the present study and other study: The Perceived Stress Scale (Cohen et al., 1983), Flourishing Scale (Diener et al., 2009).

**Pilot study**

The pilot study was conducted to produce a preliminary investigation of the association of U.S. cultural identities and well-being through self-concept clarity (see Figure S1).

**Procedure**

Data were collected through an online questionnaire recruiting participants from Prolific a crowdsourcing platform for academic research.

**Participants**

We aimed for a sample size above 300 based on recommendations regarding adequate statistical power for SEM analysis (Kline, 2015). Participants were 305 US citizens. Their mean age was 37.30 (*SD* = 13.93; all above 18 years old); and 49.5% were female, 49.2% were male, and 1.3% indicated other.

**Measurement**

Demographic information concerning age and gender was collected. Cultural identification was measured using one item for each relevant cultural stream referring to the US context (i.e., state, nation, and global). Sample item reads: “I identify with American culture”. Self-concept clarity was assessed through a proxy item from the Self-Concept Clarity Scale (Campbell et al., 1996; α=.91) tapping into the extent to which self-beliefs are clearly and confidently defined, internally consistent, and stable. The item reads: “In general, I have a clear sense of who I am and what I am”. These items were answered using a 7-point Likert scale ranging from 1 (*Strongly disagree*) to 7 (*Strongly agree*).

Life satisfaction was assessed using the Satisfaction with Life Scale (Diener et al., 1985; α = .91) tapping into global life satisfaction through five items. Sample items include, “In most ways my life is close to my ideal.” Each item was answered using a 7-point Likert scale ranging from 1 (*Strongly disagree*) to 7 (*Strongly agree*).

**Results**

The correlation matrix (Table S1) indicates positive associations among the study variables.

| Table S1. Correlation matrix and means for the Pilot Study | | | | | |
| --- | --- | --- | --- | --- | --- |
|  | 2. | 3. | 4. | 5. | *M* (*SD*) |
| 1. State identification | .57** | .39** | .35** | .40** | 4.33 (1.71) |
| 2. National identification |  | .33** | .35** | .39** | 4.58 (1.67) |
| 3. Global identification |  |  | .31** | .31** | 4.41 (1.45) |
| 4. Self-concept clarity |  |  |  | .53** | 5.49 (1.40) |
| 5. Satisfaction with life |  |  |  |  | 4.12 (1.55) |
| Note. **p*<.05; ***p*<.01 |  |  |  |  |  |

The hypothesized indirect effects model associating cultural identities with self-esteem and life satisfaction through self-concept clarity was estimated using SEM in Mplus, yielding good fit to the data, χ^2^(21) = 30.11, *p* = .090; CFI = .992; SRMR = .019; RMSEA = .038, 90%CI = [.000; .066]. Results (Figure S2) indicated positive associations of state (β = .16, *p* = .037, 95%CI = [0.10; 0.30]), national (β = .21, *p* = .007, 95%CI = [0.06; 0.35]), and global (β = .18, *p* = .004, 95%CI = [0.06; 0.30]) cultural identifications with self-concept clarity. State (β = .16, *p* = .022, 95%CI = [0.02; 0.29]) and national (β = .16, *p* = .027, 95%CI = [0.02; 0.29]) cultural identification, as well as self-concept clarity (β = .43, *p* < .001, 95%CI = [0.31; 0.55]), were positively linked with satisfaction with life, whereas global cultural identification was not associated with this aspect of well-being (β = .06, *p* = .300, 95%CI = [-0.06; 0.18]).

Evaluating the indirect effects indicated three significant paths relating state (*β* = .07, *p* = .048, 95%CI = [0.00; 0.13]), national (*β* =.09, *p* = .016, 95%CI = [0.02; 0.16]), and global (*β* =.08, *p* = .011, 95%CI = [0.02; 0.14]) cultural identification with life satisfaction through self-concept clarity.

**Preliminary Discussion**

The results of this pilot study support the assumption that regional, national, and global cultural identities are nested identities that are associated with well-being through self-concept clarity. Nevertheless, the Pilot Study was limited by (1) the use of single-item measures of cultural identification and self-concept clarity, (2) the weak effects suggesting a replication, as well as (3) a narrow conception of well-being (life satisfaction as the only aspect of well-being that was assessed). These limitations will be addressed in the following studies.

**Examining state identity**

We conducted an explorative investigation of state identity. We roughly grouped the participants from Study 2 into Woodward’s sub-national cultures (Woodward, 2012). We then employed Analysis of Variance (ANOVA) to examine mean differences between cultural identities (local, national, and global) across the sub-groups.

| Cultural group |  | State identity | National identity | Global identity | Correlation between state and national identity |
| --- | --- | --- | --- | --- | --- |
|  | *n* | *M* (*SD*) | *M* (*SD*) | *M* (*SD*) | *r* |
| Yankeedom | 106 | 4.23 (1.50) | 4.22 (1.52) | 4.12 (1.25) | .60** |
| Deep South | 103 | 4.19 (1.80) | 4.42 (1.85) | 4.41 (1.55) | .77** |
| New Netherland | 42 | 4.39 (1.30) | 4.25 (1.43) | 3.92 (1.28) | .50** |
| Tidewater | 42 | 4.07 (1.37) | 4.10 (1.48) | 4.14 (1.45) | .81** |
| Greater Appalachia | 19 | 4.42 (1.62) | 4.84 (1.38) | 4.05 (1.36) | .86** |
| Midlands | 40 | 4.91 (1.91) | 4.49 (1.73) | 4.40 (1.24) | .83** |
| New France | 7 | 4.51 (1.21) | 5.48 (1.03) | 3.29 (1.72) | -.27 |
| El Norte | 17 | 4.29 (1.55) | 4.84 (0.97) | 4.24 (1.17) | .49** |
| Far West | 54 | 4.29 (1.55) | 4.26 (1.67) | 4.30 (1.64) | .37* |
| Total | 430 | 4.27 (1.56) | 4.36 (1.61) | 4.21 (1.41) |  |
| Statistical comparison (ANOVA) |  | *F*(8, 429) = 0.401, *p* = .920, η^2^ = 0.008 | *F*(8, 429) = 1.162, *p* = .321, η^2^ = 0.022 | *F*(8, 429) = 1.065, *p* = .387, η^2^ = 0.020 |  |

We found no statistically significant difference between the nested cultural identities across these sub-national cultural groups. There were some considerable differences between the groups in regard to the correlation between state and national identity. However, some groups were so small (e.g., *n* = 7) that these results should be interpreted with great care. Accordingly, the intranational sub-groupings did not appear to include great heterogeneity vis-à-vis nested cultural identity.

**References:**

Botella, C., Molinari, G., Fernández-Álvarez, J., Guillén, V., García-Palacios, A., Baños, R. M., & Tomás, J. M. (2018). Openness to the Future Scale (OFS) *Health and quality of life outcomes, 16*, 1-16. https://doi.org/10.1037/t67333-000

Campbell, J. D., Trapnell, P. D., Heine, S. J., Katz, I. M., Lavallee, L. F., & Lehman, D. R. (1996). Self-concept clarity: Measurement, personality correlates, and cultural boundaries. *Journal of Personality* *and Social Psychology, 70,* 141-156. <https://doi.org/10.1037/0022-3514.70.1.141>.

Cohen, S., Kamarck, T., and Mermelstein, R. (1983). A global measure of perceived stress. *Journal of Health and Social Behavior,* 24, 386-396.

Diener, E., Emmons R. A., Larsen, R. J., and Griffin, S. (1985). "The Satisfaction with Life Scale." *Journal of Personality Assessment, 49*(1), 71-75. <https://doi.org/10.1207/s15327752jpa4901_13>.

Diener, E., Wirtz, D., Tov, W., Kim-Prieto, C., Choi, D., Oishi, S., & Biswas-Diener, R. (2009: New measures of well-being: Flourishing and positive and negative feelings. *Social Indicators Research, 39,* 247-266.

Hayes A. F. (2013). *Methodology in the social sciences. Introduction to mediation, moderation, and conditional process analysis: A regression-based appr*

Kline, R, B. (2015). *Principles and practice of structural equation modeling*. New York, US: Guilfort Press.

Ozer, S., & Bertelsen, P. (2019). Countering Radicalization: An Empirical Examination from a Life Psychological Perspective. *Peace and Conflict, 25*(3), 211-225. <https://doi.org/10.1037/pac0000394>.

Rosenberg, M. (1965). Rosenberg self-esteem scale (RSE). *Acceptance and commitment therapy. Measures package*, *61*(52), 18.

Woodard, C. (2012). *American nations: A history of the eleven rival regional cultures of North America*. Penguin.
